# Insights into COVID-19 data collection and management in Malawi: exploring processes, perceptions, and data discrepancies

**DOI:** 10.12688/wellcomeopenres.21131.1

**Published:** 2024-04-24

**Authors:** Amelia Taylor, Thokozani Liwewe, Jim Todd, Chisomo Kankhwali, Anne Mwale, Sylvia Kiwuwa-Muyingo

**Affiliations:** 1 Malawi University of Business and Applied Sciences, Blantyre, Malawi; 2 Lilongwe District Health Office, Lilongwe, Malawi; 3 The London School of Hygiene and Tropical Medicine, London, UK; 4 The Public Health Institute of Malawi, Lilongwe, Malawi; 5 African Population and Health Research Center, Nairobi, Nairobi County, Kenya

**Keywords:** COVID-19 data management, surveillance, form design, data quality, training, line list

## Abstract

**Background:**

The completion of case-based surveillance forms was vital for case identification during COVID-19 surveillance in Malawi. Despite significant efforts, the resulting national data suffered from gaps and inconsistencies which affected its optimal usability. The objectives of this study were to investigate the processes of collecting and reporting COVID-19 data, to explore health workers’ perceptions and understanding of the collection tools and processes, and to identify factors contributing to data quality.

**Methods:**

A total of 75 healthcare professionals directly involved in COVID-19 data collection from the Malawi Ministry of Health in Lilongwe and Blantyre participated in Focus Group Discussions and In-Depth Interviews. We collected participants’ views on the effectiveness of surveillance forms in collecting the intended data, as well as on the data collection processes and training needs. We used MAXQDA for thematic and document analysis.

**Results:**

Form design significantly influenced data quality and, together with challenges in applying case definitions, formed 44% of all issues raised. Concerns regarding processes used in data collection and training gaps comprised 49% of all the issues raised. Language issues (2%) and privacy, ethical, and cultural considerations (4%), although mentioned less frequently, offered compelling evidence for further review.

**Conclusions:**

Our study highlights the integral connection between data quality and the design and utilization of data collection forms. While the forms were deemed to contain the most relevant fields, deficiencies in format, order of fields, and the absence of an addendum with guidelines, resulted in large gaps and errors. Form design needs to be reviewed so that it appropriately fits into the overall processes and systems that capture surveillance data. This study is the first of its kind in Malawi, offering an in-depth view of the perceptions and experiences of health professionals involved in disease surveillance on the tools and processes they use.

## Introduction

Integrated Disease Surveillance and Reporting Systems (IDSRs) aim to collect health data for multiple diseases using standardized tools. Case-based surveillance involves the ongoing and rapid detection of identifiable cases for follow-up purposes, with every individual case identified being reported using a case-based form ^1^. Case-based surveillance has played a key role in managing the coronavirus disease caused by severe acute respiratory syndrome 2 (SARS-CoV-2) (COVID-19) worldwide ^2– 4^. In Malawi ^5^, case surveillance data was first collected by facilities and thereafter sent to the district levels, where IDSR focal persons, responsible for its quality, aggregated it and sent it nationally to the Ministry of Health for analysis and public dissemination. For COVID-19, cases were recorded using the Case-Based Surveillance Reporting form (CBSR) and line lists ^6^. The CBSRs were completed in hard copies, and line lists were both digital and in hard copies. In Malawi, the initial hope was to deploy several existing digital health tools to capture COVID-19 data ^7^, notably a dedicated system called One Health Surveillance Platform ^8^. However, a “COVID-19 pandemic-driven surge in demand for digital data in the health sector” coupled with challenges in digital rollout and uptake in the country meant that the anticipated use of such tools did not materialize ^9^.

The Malawi COVID-19 surveillance data necessitated great effort to collect and compile. Malawi reported regular event detection data ^10^, such as total cases, deaths, and recoveries; however, like other African countries ^11, 12^, experienced challenges in gathering, maintaining, and reporting case-based data ^13^ and comprehensive risk assessment data such as patient comorbidities and hospitalizations ^14^. Patient characterization studies ^15^ or prediction studies ^16^ of patients hospitalized with COVID-19 were performed in small cohorts, with large discrepancies being reported for the prevalence of disease between small seropositivity studies and nationally reported numbers ^14^. In addition, difficulties in producing national individual-level COVID-19 data that was timely and accurate led to bottlenecks in data sharing among stakeholders, which, in some cases, was exacerbated by publication timelines of research projects competing for access to the same data ^17^. Although steps were taken by the Public Health Institute of Malawi (PHIM) and IDSR offices to achieve good data quality, many issues remained unsolved throughout the pandemic. Most of the data quality issues were detected late, leading to a time-consuming root cause analysis process to understand the sources of data discrepancies coming upstream from the district and from the facility level where the IDSR forms were completed.

## The COVID-19 IDSR case base surveillance form

COVID-19 was a new disease. The IDSR processes needed to be adapted quickly throughout the data collection period to address changes in case definitions and operational demands. In Malawi, swift efforts were made to adapt the Malawi IDSR guidelines ^18^ and CBSR forms to incorporate variables from the COVID-19 World Health Organization Case Reporting Form ^19^. Although other forms were used for specific purposes (e.g., at entry ports, laboratory logbooks, and data aggregation forms), the CBSR served as the primary instrument for epidemiological data collection on COVID-19 nationally and was required to be filled in for all individuals testing for COVID-19 in both government and private healthcare facilities. The purpose of the form was to collect data on suspected and confirmed cases and captured several types of data: clinical manifestations (i.e., symptoms and underlying conditions), laboratory results and epidemiological information (e.g., person, place, and time), and specific behaviors (e.g., travel, clustering, contact with suspected cases), as well as levels of certainty (e.g., confirmed/suspect). The form was structured in sections to be filled in by different cadres working in collaboration with each other: health surveillance assistants (HSAs) / environmental surveillance officers (EHOs), clinicians, and laboratory technicians.

A national list of COVID-19 cases was compiled from district-level line lists every week. The line list was designed to collect data obtained through CBSR and hospital notes. The line list included additional information on hospitalization, treatment, case outcomes, and contact tracing data. Some of this data had to be collected retrospectively, for example, patient outcomes. Observations from our preliminary analysis of the line-list data led us to believe that there was a need to further investigate both the CBSR design and its utilization for COVID-19 data collection. We noted gaps and errors on the line lists that could be traced to data collection and data merging at the district level. Our observations served as the initial catalyst for this study.

## Purpose

To improve the understanding of COVID-19 data collected in Malawi through the two main data collection instruments, the CBSR, and the line list, we conducted a study in Lilongwe and Blantyre. These cities registered 80% of all positive COVID-19 cases in Malawi. This study aimed to gain insights into the operational aspects of COVID-19 data management, understand health worker perspectives on surveillance data, and identify factors influencing data accuracy and consistency within the district's reporting system.

Previous studies have examined the structure of the IDSR system in Malawi looking into the quality of reported aggregated numbers ^5^. A recent scoping review highlighted operational challenges and underscored the complexity of Malawi’s data surveillance landscape ^20^. Our study is the first to look in depth at the use of case-based surveillance forms in Malawi and provides an indirect measure of “content validity” ^21^: how well the data collected via the form cover the actual area of investigation. In this respect, we are looking at how the format and structure of the CBSR and its use could have led to gaps or errors in the data. We focused on context and purpose and adopted a qualitative approach. In this respect, our work is a study of the lived experiences of clinicians, laboratory technicians, surveillants, and health officers who used the form and other tools for collecting COVID-19 data.

## Methods

### Setting and study population

The study focused on capturing the experiences of individuals in various roles who actively engaged in COVID-19 data collection, namely, laboratory technicians, environmental health officers, clinicians, and health surveillance assistants. Interviews were conducted with 4 key cadres from the Malawi Ministry of Health who were involved in the supervision of surveillance activities for COVID-19. We conducted nine face-to-face Focus Group Discussion (FGD) sessions ^22^ in Lilongwe and Blantyre. Participants were selected strategically from key locations that registered high numbers of COVID-19 cases. In Lilongwe, our study encompassed five FGD sessions strategically held at the Bwaila Hospital and Area 25 District Health Office (DHO). These locations were chosen due to their significance, with Bwaila Hospital serving as a focal point where all district case-based surveillance forms were submitted and compiled into line lists for transmission to PHIM by the IDSR coordinator. Similarly, in Blantyre, our investigation covered the District Health Office, recognizing its critical role in the region.

### Study design

We used a case study approach to qualitative inquiry to help us understand the experiences of key personnel involved in COVID-19 surveillance by filling in CBSRs and reporting data through line-lists. The methods were one-to-one interviews and focus group discussions.

The FGD group sizes ranged between 6 and 10 participants. Each lasted an average of one hour except for the FGD at Bwaila, which was longer. Participants were grouped by cadre to ensure that they had common ground for meaningful discussions while also allowing for diverse experiences and facilitating debates or differences of opinion. FGDs were moderated using a structured topic guide. The moderator used 8 open-ended questions and additional probes, such as “Please give an example” or “Please explain in more detail”, to clarify the answers and deepen the discussion. The co-moderator assisted in digitally audio recording the focus group discussions, observing group interactions, and taking notes. All sessions were recorded. Prior to the discussions, all participants were thoroughly briefed about the scope of the session. Participants signed an informed consent form. We also requested that each participant completed a brief questionnaire and provided specific information about their professional background, that was particularly relevant to COVID-19 data collection.

Four interviews were conducted with key informants, each lasting an average of 40 min. From the Environmental Surveillance and Response team, we interviewed the main officer overseeing the collection, entry, and usage of case-based surveillance forms in facilities. At the district health office, we interviewed the leader responsible for overseeing the IDSR coordinators’ duties, and for maintaining feedback channels from PHIM to districts regarding data quality. We interviewed the national lead from the clinical team. Finally, we interviewed the head of the Bwaila laboratory who was overseeing the laboratory team working on COVID-19 tests. The interviews looked into processes for collecting, aggregating, and analyzing data at national level. All interviews were recorded; 3 were held over the phone, and one was held in person.

To complement our qualitative study, document analysis was used. We consulted technical guidelines to determine the role of each cadre involved in COVID-19 surveillance. We performed a small audit of completed CBSR forms to assess the extent of data completeness and identify problematic fields.

### Data collection

Through the questionnaire we collected quantitative data from FGD participants, such as their position, age, gender, and type of involvement in COVID-19 surveillance: period of involvement in terms of the pandemic years 2020, 2021, 2022, 2023; locations where they participated in surveillance, e.g. schools, points of entry, clinics, and the type of training they received on the use of CBSR for COVID-19 surveillance.

From FGDs and interviews, we collected qualitative data in the form of audio recordings, and written notes taken by co-moderators during FGD sessions and interviews. Data was collected over three days in Lilongwe and over two days in Blantyre.

From document analysis and form audit, we collected semi-quantitative results. We obtained a table of roles and responsibilities for data collection of key cadres involved in COVID-19 surveillance. The CBSR audit involved examining physical evidence and gathering statistics on the completeness of the forms. This information helped us further understand the feedback from participants in FGDs and interviews regarding fields such as test results, and case outcomes that were consistently left empty.

## Ethical clearance

The study was part of a project conducted in close collaboration with the Ministry of Health through the Public Health Institute of Malawi. The scope of the project was to understand the national COVID-19 data collected via IDSR surveillance in Malawi. The project was sponsored and approved by the Malawi National Health Sciences Research Committee protocol #21/03/2669 issued on 11th March 2021 and renewed on 4th March 2022. FGDs and interviews were approved by local research committees at the Lilongwe District Health Office on 2nd May 2023 and the Blantyre District Health Office on 24th July 2023. Participants were required to provide written informed consent; this was collected by trained facilitators. All data and documents were kept in the custody of the principal investigator in a secure and restricted location.

## Data analysis

The statistics derived from the quantitative data were generated using Excel. These provide a profile of our participants in terms of demographics such as age and gender, and their experience and training in COVID-19 surveillance.

We employed thematic content analysis to scrutinize the data collected via FGDs. The audio recordings were professionally transcribed. We coded the transcripts using the software program MAXDQA ^23^. The use of MAXDQA allowed us to manage all our resources in one place, to define and apply codes efficiently to paragraphs and sections of documents, and to obtain insights into the data based on applied codes. MAXDQA is a paid software, but several free alternatives exist, the closest in terms of features to MAXDQA being QualCoder ^24^. The resulting themes were organized into categories. We adhered to a systematic analysis approach, ensuring the inclusion of all recordings and transcriptions in their entirety. Codes were affixed to data segments, each segment corresponding to one response from a participant. These codes served as descriptive labels for the issues expressed within a segment. We initiated the process by assigning codes for the following categories: (a) form structure and element issues, (b) process challenges, and (c) training issues. Form structure and element issues dealt primarily with form design and encompass concerns related to the layout, arrangement, logical succession of elements, missing options, and clarity of elements on the form, including the use of abbreviations. Processes and training included issues concerning training and processes for obtaining, recording, and transmitting data, including coordinating work among the different cadres. Three new categories emerged from our analysis: (d) Knowledge of and applications of clinical case definitions; (e) Privacy, ethical, and cultural considerations; and (f) Language issues. Case definition knowledge and application specifically address how well the form captures the clinical case definition for COVID-19 cases. Privacy, ethical, and cultural considerations refer to the use of the form and its associated processes in an ethical way, that respects the privacy of respondents and data recorders and is culturally sensitive. Language issues pertained to ambiguous or confusing as well as communication challenges that could have influenced the quality of the data collected through the form.

Interview transcripts were taken through an exploratory analysis to extract insights into processes for data collection and data flow within the IDSR system, including aspects of training. The CBSR audit was used to derive some representative statistics on the type of cadres who completed data on forms, and the number of fields that were frequently empty.

## Results from FGDs

A total of 9 FGDs and four one-on-one interviews were conducted in Lilongwe ( Table 1) ^6^. There were 35 males and 36 females. Among the participants in Lilongwe, 24 were female and 17 were male; in Blantyre, 18 were male and 12 were female. Most participants were in the younger age group (25–34 years). There were 23 laboratory officers, 17 health surveillance assistants, 19 environmental health officers, and 12 clinicians (10 clinical officers and 2 medical assistants). All participants were involved in COVID-19 data collection over the years between 2020 and 2023 at a variety of locations other than health facilities. More than half conducted targeted testing at organizations such as banks or at points of entry. More than a quarter of the participants were involved in mass testing at schools and prisons.

**Table 1.** Recruitment and characteristics of the participants in the nine focus groups.

*Cadre*	*FGDs in Lilongwe*	*FGDs in Blantyre*	*Total*
***Total Number of Participants***	41	30	71
***Type of cadres participating***			
*Clinical Officers*	7	5	12
*Health Surveillance Officers*	8	9	17
*Environmental Health Officers*	10	9	19
*Laboratory Technicians*	16	7	23
***Sex***			
*Female n (%)*	24 (59%)	12 (40%)	36 (51%)
*Male n (%)*	17 (41%)	18 (60%	35 (49%)
***Age ranges***			
*Under 25*	3	1	4
*25–34*	20	11	31
*35–44*	11	12	23
*45–54*	4	3	7
*55 and over*	3	3	6
***Years of Participation in COVID-19 data collection, n***			
*2020 only*	1	4	7
*2021 only*	1	4	9
*2022 only*	0	1	11
*More than one year*	8	12	11
*All Years*	20	19	39
***Type of training received (if any)***			
*On the job*	21	4	25
*Orientation*	2	10	12
*No training*	18	16	34
***Location where participants were involved in data collection, n***			
*Health Facility*	27	29	56
*Private Institutions*	20	24	44
*Ports of Entry*	8	22	30
*Schools*	20	18	38
*Prisons*	13	17	30
*All types*	5	11	16
***Highest Number on average of cases participants filled each week, n***			
*10–50*	12	11	23
*50–100*	15	9	24
*100–200*	6	5	11
*200–500*	7	5	12

### Participation in discussions

The rate of participation was good in all groups, and all participants contributed to discussions ( Table 2). We employed an experienced moderator to conduct all the FGDs. An analysis of the interaction between the moderator and participants ( Table 3) showed that the moderator asked all the intended questions in the nine groups and was balanced in stimulating the conversation through questioning and prompting. Most responses ( Table 4) in the FGDs focused on Form Structure and Elements Issues and Processes and Training Challenges (82%). Participants' overall impression was that the case-based surveillance form is comprehensive but demanding, especially during peak periods of COVID-19 when screening numerous clients ( Table 5).

**Table 2.** Participation in FGDs in terms of the number of contributions (responses) per participant.

*Participant * *contributions in * *segments, n*	*BLANTYRE*				*LILONGWE*				
*FGD*	*FGD1.1*	*FGD1.2*	*FGD1.3*	*FGD1.4 *	*FGD2.1*	*FGD2.2*	*FGD2.3*	*FGD2.4*	*FGD2.5*
***P1***	*9*	*15*	*9*	*3*	*18*	*5*	*6*	*6*	*4*
***P2***	*12*	*5*	*7*	*13*	*8*	*7*	*11*	*9*	
***P3***	*11*	*8*	*13*	*11*	*7*	*11*	*3*	*3*	*8*
***P4***	*9*	*7*	*7*	*10*	*9*	*15*	*6*	*5*	*2*
***P5***	*6*	*2*	*14*	*8*	*7*	*14*	*11*	*9*	*2*
***P6***		*9*	*12*	*7*	*4*	*4*	*7*	*5*	*7*
***P7***		*3*	*4*	*6*	*5*	*2*	*3*	*8*	*6*
***P8***		*16*	*7*			*3*	*6*	*6*	*8*
***P9***		*5*	*15*			*8*			
***P10***		*9*				*7*			
***Total responses***	*47*	*79*	*88*	*58*	*58*	*76*	*53*	*51*	*37*
***Participants***	*5*	*10*	*9*	*7*	*7*	*10*	*8*	*8*	*8*

*Note*. *FGD1.1=Clinicians (Blantyre) FGD1.2=Environmental Health Officers (Blantyre) FGD1.3 Health Surveillance Assistants (Blantyre) FGD1.4 = Laboratory Technicians (Blantyre) FGD2.1= Clinicians (LL Bwaila) FGD2.2=Environmental Health Officers (LL Bwaila) FGD2.3=Health Surveillance Assistants (LL Area 25 HC) FGD2.4=Laboratory Technicians (LL Area 25 HC) FGD2.5=Laboratory Technicians (LL Bwaila) Participants are labeled in the same way, P1, P2 and so on. However, there was no relationship between P1 in one group and P1 in the other group. All groups had distinct participants. Participant P2 in FGD2.5 had to leave the meeting soon after joining for a family emergency.*

**Table 3.** FGD moderation and structure.

		*BLANTYRE*				*LILONGWE*						
		*FGD1.1*	*FGD1.2*	*FGD1.3*	*FGD1.4*	*FGD2.1*	*FGD2.2*	*FGD2.3*	*FGD2.4*	*FGD2.5*	*Total*	*% Total*
	**Segments being participants' responses, n**	**> *47***	**> *79***	**> *88***	**> *58***	**> *58***	**> *76***	**> *53***	**> *51***	**> *37***	**> *547***	**> *69%***
	**Segments being questions and prompts, n**	**> *23***	**> *30***	**> *36***	**> *29***	**> *23***	**> *27***	**> *24***	**> *28***	**> *22***	**> *242***	**> *31%***
**Segments being questions and prompts breakdown**	*Questions soliciting participation*	*8*	*9*	*7*	*7*	*7*	*11*	*9*	*5*	*6*	*69*	*9%*
	Questions on form design*	*16*	*14*	*19*	*14*	*10*	*13*	*14*	*16*	*12*	*128*	*16%*
	*Overall Perception*	*3*	*1*	*2*	*1*	*2*	*4*	*3*	*3*	*2*	*21*	*3%*
	*Form structure and elements*	*3*	*4*	*7*	*3*	*3*	*4*	*2*	*2*	*5*	*33*	*4%*
	*Difficult or unclear elements*	*4*	*5*	*4*		*1*	*2*	*4*	*6*	*3*	*29*	*4%*
	*Challenges when collecting data*	*6*	*4*	*6*	*5*	*4*	*3*	*5*	*5*	*1*	*39*	*5%*
	Questions on processes*	*2*	*6*	*10*	*6*	*6*	*2*	*2*	*6*	*4*	*44*	*6%*
	*Coordination among cadres*	*0*	*4*	*3*				*1*	*3*		*11*	*1%*
	*Questions about seeking clarity*	*2*	*2*	*4*	*2*		*2*	*2*	*2*	*1*	*17*	*2%*
	**Questions on data discrepancies**	*2*	*3*	*3*	*6*	*2*	*3*	*2*	*2*	*3*	*26*	*3%*
	**Questions about the line list (EHOs)**	*0*	*4*	*0*	*0*	*0*	*5*	*0*	*0*	*0*	*9*	*1%*
	**Questions on training**	*2*	*1*	*2*	*2*	*2*	*2*	*2*	*2*	*0*	*15*	*2%*
	**Questions on recommendations**	*1*	*2*	*2*	*1*	*3*	*2*	*4*	*2*	*3*	*20*	*3%*
	**Ratio of Questions to responses**	**32%**	**27%**	**33%**	**38%**	**28%**	**21%**	**28%**	**45%**	**43%**		
	**Text Coverage of Interviewer's Questions**	**11%**	**12%**	**16%**	**18%**	**14%**	**7%**	**10%**	**16%**	**13%**		

		*BLANTYRE*				*LILONGWE*						
		*FGD1.1*	*FGD1.2*	*FGD1.3*	*FGD1.4*	*FGD2.1*	*FGD2.2*	*FGD2.3*	*FGD2.4*	*FGD2.5*	*Total*	*% Total*
	**Segments being participants' responses, n**	*47*	*79*	*88*	*58*	*58*	*76*	*53*	*51*	*37*	*547*	*69%*
	**Segments being questions and prompts, n**	*23*	*30*	*36*	*29*	*23*	*27*	*24*	*28*	*22*	*242*	*31%*
**Segments being questions and prompts breakdown**	Questions on form design*	*16*	*14*	*19*	*14*	*10*	*13*	*14*	*16*	*12*	*128*	*16%*
	*Overall Perception*	*3*	*1*	*2*	*1*	*2*	*4*	*3*	*3*	*2*	*21*	*3%*
	*Form structure and elements*	*3*	*4*	*7*	*3*	*3*	*4*	*2*	*2*	*5*	*33*	*4%*
	*Difficult or unclear elements*	*4*	*5*	*4*		*1*	*2*	*4*	*6*	*3*	*29*	*4%*
	***Challenges when collecting data***	*6*	*4*	*6*	*5*	*4*	*3*	*5*	*5*	*1*	*39*	*5%*
	Questions on processes*	*2*	*6*	*10*	*6*	*6*	*2*	*2*	*6*	*4*	*44*	*6%*
	*Coordination among cadres*	*0*	*4*	*3*				*1*	*3*		*11*	*1%*
	*Questions about seeking clarity*	*2*	*2*	*4*	*2*		*2*	*2*	*2*	*1*	*17*	*2%*
	**Questions on data discrepancies**	*2*	*3*	*3*	*6*	*2*	*3*	*2*	*2*	*3*	*26*	*3%*
	**Questions about the line list (EHOs)**	*0*	*4*	*0*	*0*	*0*	*5*	*0*	*0*	*0*	*9*	*1%*
	**Questions on training**	*2*	*1*	*2*	*2*	*2*	*2*	*2*	*2*	*0*	*15*	*2%*
	**Questions on recommendations**	*1*	*2*	*2*	*1*	*3*	*2*	*4*	*2*	*3*	*20*	*3%*
	**Ratio of Questions to responses**	**49%**	**38%**	**41%**	**50%**	**40%**	**36%**	**45%**	**55%**	**59%**	**44%**	
												
	**Text Coverage of Interviewer's Questions**	**11%**	**12%**	**16%**	**18%**	**14%**	**7%**	**10%**	**16%**	**13%**		

**Table 4.** Frequencies of the top-level categories in all FGDs.

*Category*	*Number of issues raised in each* *category from participants' responses*	*% Total*
***Total in all categories***	*978*	*100%*
*Form Design Issues*	*320*	*33%*
*Processes Challenges*	*297*	*30%*
*Training Issues*	*189*	*19%*
*Clinical Case Definitions Knowledge and Application*	*108*	*11%*
*Privacy, Ethical, and Cultural Considerations*	*44*	*4%*
*Language Issues*	*20*	*2%*

**Table 5.** Details of issues raised during FGDs.

	Issues / No. issues per FGD (count)		FGD1.1	FGD1.2	FGD1.3	FGD1.4	Blantyre	FGD2.1	FGD2.2	FGD2.3	FGD2.4	FGD2.5	Lilongwe	Total
**Clinical Case** **Definitions Knowledge** **and Applications**	Challenges in recording underlying conditions		3%	16%	15%	9%	43%	18%	19%	9%	9%	1%	**57%**	**100%**
	Challenges in recording symptoms		0%	10%	15%	5%	29%	39%	20%	5%	0%	7%	**71%**	**100%**
	**Subtotal**		**2%**	**14%**	**15%**	**7%**	38%	26%	**19%**	**7%**	**6%**	**4%**	**62%**	**100%**
**Form Structure and Elements**	Challenges in recording demographic data		12%	19%	23%	7%	61%	2%	19%	14%	4%	0%	**39%**	**100%**
	Length, fields order, layout and font size issues		10%	31%	13%	13%	67%	8%	2%	10%	6%	6%	**33%**	**100%**
	Fields unclear how to fill in		18%	21%	26%	6%	71%	0%	6%	0%	12%	12%	**29%**	**100%**
	Client unwillingness to give information		0%	14%	18%	11%	43%	14%	29%	7%	7%	0%	**57%**	**100%**
	Vaccination data issues		20%	4%	12%	12%	48%	8%	16%	8%	16%	4%	**52%**	**100%**
	Fields did not correspond to processes		13%	8%	21%	8%	50%	0%	21%	0%	13%	17%	**50%**	**100%**
	Addendum on how to fill in the form not provided		5%	16%	16%	11%	47%	11%	21%	11%	11%	0%	**53%**	**100%**
	Fields with inadequate options		11%	26%	16%	11%	63%	0%	21%	0%	11%	5%	**37%**	**100%**
	Ambiguous wording and lack of guidance		6%	39%	6%	0%	50%	6%	6%	11%	0%	28%	**50%**	**100%**
	Timeline of the form		15%	15%	15%	0%	46%	0%	31%	0%	15%	8%	**54%**	**100%**
	Unfamiliar abbreviations or terms		8%	42%	25%	0%	75%	0%	0%	0%	0%	25%	**25%**	**100%**
	Case outcome and test results		18%	0%	18%	9%	45%	0%	27%	0%	18%	9%	**55%**	**100%**
	Repetitive fields		0%	29%	0%	14%	43%	0%	0%	43%	14%	0%	**57%**	**100%**
	Logical connection between fields		0%	40%	0%	0%	40%	0%	20%	20%	20%	0%	**60%**	**100%**
	**Subtotal**		**11%**	**21%**	**17%**	**8%**	**57%**	**4%**	**15%**	**8%**	**9%**	**7%**	**43%**	**100%**
	**Language Issues**		**0%**	**5%**	**5%**	**0%**	**10%**	**45%**	**25%**	**10%**	**0%**	**10%**	**90%**	**100%**
**Privacy, Ethical, Cultural** **Considerations**	Privacy and inadequate space		0%	0%	12%	6%	18%	0%	47%	24%	0%	12%	**82%**	**100%**
	Rumours, misconceptions and myths		10%	20%	30%	0%	60%	20%	20%	0%	0%	0%	**40%**	**100%**
	Sensitive questions		10%	20%	10%	10%	50%	0%	20%	10%	0%	20%	**50%**	**100%**
	Consent Issues		0%	0%	0%	33%	33%	33%	0%	0%	33%	0%	**67%**	**100%**
	Fear of stigma		33%	0%	0%	0%	33%	0%	67%	0%	0%	0%	**67%**	**100%**
	Cultural concerns (e.g. burial traditions)		0%	0%	0%	0%	0%	100%	0%	0%	0%	0%	**100%**	**100%**
	**Subtotal**		**7%**	**9%**	**14%**	**7%**	**36%**	**9%**	**32%**	**11%**	**2%**	**9%**	**64%**	**100%**
**Process Challenges**	**Process** **Ambiguity (97)**	Issues in transmission of test results	20%	7%	9%	7%	43%	2%	27%	0%	16%	11%	**57%**	**100%**
		Issues in contact tracing	17%	21%	28%	7%	72%	0%	17%	7%	0%	3%	**28%**	**100%**
		Process ambiguity (other)	0%	17%	0%	17%	35%	0%	43%	9%	13%	0%	**65%**	**100%**
		Form inadequate to capture reinfection	0%	0%	0%	0%	0%	0%	0%	0%	0%	100%	**100%**	**100%**
	Reporting / transmission / linkage with national systems		14%	10%	8%	19%	51%	0%	15%	7%	4%	22%	**49%**	**100%**
	Challenges in work coordination among cadres		19%	11%	9%	5%	44%	4%	11%	21%	9%	12%	**56%**	**100%**
	Data quality issues due to workload or time constraints		14%	19%	10%	7%	50%	2%	7%	2%	10%	29%	**50%**	**100%**
	Logistics and Resource Constraints		12%	12%	12%	19%	54%	8%	8%	12%	4%	15%	**46%**	**100%**
	Use of three copies of the form		0%	0%	67%	33%	100%	0%	0%	0%	0%	0%	**0%**	**100%**
	**Subtotal**		**15%**	**12%**	**11%**	**12%**	**50%**	**2%**	**16%**	**8%**	**8%**	**15%**	**50%**	**100%**
**Training Issues**	**Inadequate or lack of training (145)**	Inadequate or lack of training	10%	23%	15%	8%	56%	11%	8%	7%	5%	13%	**44%**	**100%**
		Value of multidisciplinary teams	26%	5%	21%	11%	63%	0%	0%	11%	5%	21%	**37%**	**100%**
		On the job training	14%	0%	0%	21%	36%	14%	14%	0%	7%	29%	**64%**	**100%**
		Use of intuition when filling in data	13%	38%	0%	0%	50%	0%	13%	13%	13%	13%	**50%**	**100%**
		Training on case definitions	0%	0%	50%	0%	50%	33%	17%	0%	0%	0%	**50%**	**100%**
		Lack of understanding how the data will be analysed	17%	0%	0%	0%	17%	50%	33%	0%	0%	0%	**83%**	**100%**
		Data entry / transcription issues	0%	33%	0%	0%	33%	0%	33%	0%	0%	33%	**67%**	**100%**
		Use of shortcuts	50%	0%	0%	0%	50%	0%	0%	0%	0%	50%	**50%**	**100%**
		**Subtotal**	**12%**	**17%**	**14%**	**8%**	**52%**	**11%**	**10%**	**6%**	**5%**	**16%**	**48%**	**100%**
	Misconceptions / attitudes about the data collected		6%	17%	6%	6%	33%	33%	17%	6%	11%	0%	**67%**	**100%**
	Questions that could not be answered by patients		18%	12%	18%	12%	59%	6%	12%	6%	12%	6%	**41%**	**100%**
	Incorrect data capturing of key demographics information		0%	14%	0%	0%	14%	0%	43%	14%	0%	29%	**86%**	**100%**
	Other training issues		50%	0%	0%	0%	50%	50%	0%	0%	0%	0%	**50%**	**100%**
	**Subtotal**		**12%**	**16%**	**13%**	**8%**	**49%**	**13%**	**12%**	**6%**	**6%**	**14%**	**51%**	**100%**
	**Total**		**11%**	**16%**	**14%**	**9%**	**49%**	**9%**	**16%**	**8%**	**7%**	**11%**	**51%**	**100%**

**Table 6.** Cadres’ responsibilities in COVID-19 data collection.

Cadres	Type of Processes/ Characteristics	Description
**Health Surveillance Assistants**	Main responsibilities	Screening patients/clients at health facility, border points, recording temperatures, asking COVID-19 related questions, providing health talks, crowd control, enforcing preventive measures, and filling case-based forms.
	Type of surveillance data	Data on CBSR and health declaration forms.
	Frequency of data collection	Daily interactions with clients at the point of service or at points of entry.
	Coordination with other cadres	Supervised by Environmental Health Officers and Port Health Officer (airport team), work with laboratory officers and Clinicians.
	Data entry into surveillance systems	Not involved in data entry except for the airport team, which produces a report on COVID-19 screening and testing services.
	Data quality involvement	Not involved in data quality issues except at the point of data generation.
**Environmental Health Officers**	Main roles and responsibilities	Responsible for surveillance activities, data generation, aggregation for district reporting, contact tracing, screening, follow-up.
	Type of surveillance data	Handle forms for different activities, including CBSR, collect laboratory samples accompanying CBSR, and the issuing of discharge certificates.
	Frequency of data collection	Majority of data generated daily, especially case-based forms.
	Coordination with other cadres	Work hand in hand with Health Surveillance Assistants, clinicians, laboratory officers for surveillance activities and data collection.
	Data entry into surveillance systems	Electronic data entry is infrequently done; CBSR forms are sent via WhatsApp or email to the district for entry onto the line list.
	Data quality involvement	Data quality check/monitoring as middle-level managers; review data prior to submission to the next level.
**Clinicians**	Main roles and responsibilities	Screen and assess the eligibility of suspected patients. Refer patients to the laboratory for Covid-19 tests. Work with laboratory and surveillance team to ensure contact tracing and adherence to quarantine protocols.
	Type of surveillance data	CBSR sections for clinical data: patient presentation, establish underlying conditions and identifying risk factors. Provide data on admissions, treatments, and case outcomes at discharge to the IDSR coordinator.
	Frequency of data collection	Data is obtained from each patient, potentially on a daily basis. Regular reporting of patient outcomes and discharges.
	Coordination with other cadres	Coordinate with nurses and physiotherapy officers in clinical care; with laboratory officers for surveillance data. Constant interaction with the surveillance team for investigations and part of rapid response teams at district and facility levels.
	Data entry into surveillance systems	Clinicians document paper-based patient records, and data clerks capture this data electronically.
	Data quality involvement	Data quality issues arise during generation. Clinicians' generated data goes to the line list, where it can later be checked and verified.
**Laboratory Technicians**	Main roles and responsibilities	Conduct tests on suspected cases. Provide guidance on patient discharge during isolation monitoring.
	Type of surveillance data	Generate data on the CBSR sometimes filling in the entire form. Submit daily testing summaries to the district. Include data on tests and re-tests after isolation.
	Frequency of data collection	Daily data entry for CBSR based on suspected cases. Daily reports of COVID-19 tests carried out by the health facility.
	Coordination with other cadres	Handle testing requests from clinicians and provide results. Provide testing results to surveillance officers through CBSR and testing result slips. Follow up when client results are missing from records.
	Data entry into surveillance systems	Not involved in data entry. Use log and registers to enter data for each tested case.
	Data quality involvement	Yes but checks are not integrated in their regular activities.

## Form design: structure and elements (33%)

Participants shared concerns related to the length of the form (over 51 fields), its condensed layout, and the logical flow in the order of some of its elements (15%). Challenges in recording demographic data were prevalent. These typically surrounded the format of dates, the recording of addresses or nearby landmarks, telephone numbers, and identification documents. There was a lack of guidance for filling in dates or for recording physical addresses and landmarks and the space was inadequate:

*“The field which was enquiring about the “Physical Address” had little space to comprehensively capture the clients’ data so that another person should be able to trace the client easily”. (Clinicians)*
*“The fields were left blank. For example, the client’s phone number. If he or she didn’t have a phone, we could not write anything. On the “Physical address,” some clients could not give clear details. Furthermore, on the “Vaccinations,” other clients didn’t carry their cards along, and other clients were not explaining clearly. So, it was difficult for us to fill in such fields; hence, they were left blank.” (Clinicians)*
*“Just like what Participant One has said; on the “Demographics,” we were not asking lot of things. If the client has given us a phone number, then we could just leave out the rest of the fields. That is because the clients were too many. If the client has given me his or her phone number, then ‘What is the use of the physical address?’” (EHO)*
*“Although, as my colleagues have already said, it was time consuming to fill all the details. Some clients were giving false information. For instance, on the “landmarks,” they couldn’t tell the exact place. They could give you wrong phone numbers”. (Clinicians)*
*“I just want to add on the field where we were writing the clients first name and last name. We were making errors, in terms of the spellings. For example, the clients name is “Gift” the client would request that it be written in Chichewa as “Gifiti.” Further, they could also give us the name of a physical address that was difficult to trace. Furthermore, on the Reporter Name field; we were writing the name and the phone number. So, when we have written the phone number; sometimes, like when we went to Maula prison, some clients were calling us.” (EHO)*

The forms did not come with an addendum. While most fields were familiar to health professionals who used a similar surveillance form for cholera, this was the first time when the CBSRs were used on a such large scale. The client’s unwillingness to provide information was a contributing factor to data gaps, especially during the first waves when COVID-19 was poorly understood.

*“Clients were not willing for you to visit them in their homes. They were also denying you permission to screen their family members. Most of them would refuse. Further, most of the time the clients were giving us false physical addresses and phone numbers.” (EHO)*
*“Of course, here we are talking about Covid-19 surveillance. However, this case-based surveillance form included other health conditions. So, it might be challenging for someone to fill. For example, when you look at the “specimen collection field”; there are different types of specimens to be collected. So, if someone is not well trained on how to fill this form, one might tick on the wrong space instead of where he or she is supposed to fill.” (EHO)*
*“I should agree with participant one. The form was supposed to have a footer below, explaining the abbreviations that are on the form. (…) maybe it is not everyone who was filling out the form could understand what the form was enquiring. That is why some of the variables were not being filled. In addition, it is also possible that we were just scribbling on some of the variables.” (EHO)*

There was also an element of lack of appreciation or understanding of the importance of some of the data that was collected. For example, patients questioned the need for recording occupation or even pregnancy status or trimester. EHOs, HSAs, or Laboratory officers often did now know the reasons themselves and did not feel that they could put pressure on clients to provide these details. Such fields would then remain empty on most forms. As the CBSR was also used to support manual contact-tracing activities, issues in recording demographic data resulted in the inability of authorities to trace or monitor infections in communities.

*“I just wanted to emphasize what Participant Two and Participant One have said about the issues of training. To say that you take ownership of the thing if you have been oriented about it. We were discussing the issue of leaving gaps in case-based surveillance forms. That is because we didn’t know what errors would come up as a result of leaving gaps in some of the fields of the form. We were just saying, “At least I have filled some of the fields. I don’t know the need to fill the other fields.” So the trainings are really needed.” (Clinician)*

Other fields prone to incompleteness or inaccuracies included information about underlying conditions, and vaccinations, as well as final test results and case outcomes (29%). Participants highlighted challenges in filling in the "Type of test performed" and "Results". For laboratory-based testing, the time taken to produce test results meant that these fields were often incomplete. The delay in test results also complicated the process of matching results to the correct test dates or communicating results to clients. Similar issues were present in the line list, particularly with the "Date of outcome" field. Tracking recovery or mortality was impossible because the information was often incomplete and lacked specifics, such as the client's name and the date of death.

*“Particularly, on the last part of the form; where it says, “Case final outcome,” and “Case final classification.” I think that those fields were mostly just left blank. The clients were found “Alive and Confirmed.” However, at the end of the day, during that particular time, you could find that the client is coming to check his or her results 48 hours later. Thereafter, you gave the client his or her results. You were just going into the “LAMIS” to check the data that has come from the Laboratory. So you were just taking that data and then filling it in the “District Modulated Form.” Which we were giving the client. You were not going back to the case-based surveillance form to fill in the lower fields. It was like that. If a client has come to get his or her results; especially the “DNA/PCR” results; you were just getting the results sheet sent to you through “WhatsApp” by the Laboratory Technicians. Thereafter, you just get the result sheet and then tell the client his or her results. We were not going back to the case-based surveillance form. To say, “Where is the form? I should indicate that the client is alive and confirmed.” We were filling out this form on the first day we were screening the client. So, for the results that have come out instantly, like the results for the “Rapid Tests,” the Laboratory technician recorded whether positive or negative; still, the field “Final case outcome” was left blank.” (Clinician)*

A specific field whose logical order caused notable confusion was the Date of Onset for symptoms. This appeared separated from the symptoms section. Multiple symptoms were being recorded in the same form, so it was unclear to which of those the date of onset referred to. A similar logical problem was observed for the field recording the number of vaccination doses: it was not clear to what vaccine it referred to as the form mentioned several types of vaccines. Additionally, clients could not recall the date of the last vaccination. In all these situations, these fields remained empty, or were difficult to interpret.

## Clinical case definitions, knowledge, and applications (11%)

Participants faced challenges in recording "underlying conditions" due to the use of abbreviations such as "DM" and "COPD". Some personnel left this field blank, citing unfamiliarity with certain conditions. HSAs or EHOs do not have a formal education equal to that of a clinician. Their training is limited to an enrolment course and occasional short training ^25^. Participants suggested that the Presenting Symptoms field should allow one to document what clients report rather than going through a list of suggested predefined symptoms.

*“Some of us could have an opportunity to ask a Clinician at the facility. If he or she is not available, then we were just skipping some of the fields. For example, the field that has to do with Underlying Conditions.” (HSA)*
*“On the field Underlying conditions: They were supposed to translate some of these abbreviations. That is because most of the time you were asking the client in Chichewa. So when you say, “DM,” “what is DM?” If a person does not know what “DM” is, he or she will just skip it. Then let’s say, “Cardiovascular DZ,” “Neurological DZ,” we do not know what that is. Yes, maybe it might mean a disease, or perhaps it does not even mean a disease. Therefore, we should know what the abbreviation is saying.” (HSA).*

Clients found it difficult to differentiate between symptoms such as chest pain and shortness of breath. This sometimes led to inaccurate information being recorded. There were also instances where false information was recorded unintentionally. For various reasons, patients might exaggerate symptoms, leading to discrepancies between reported symptoms and clinical observations. Situations when clients were asymptomatic but tested positive challenged the form's applicability to all situations. Sometimes as the form filling progressed, it became obvious that clients provided inaccurate answers to previous questions. Some customers only acknowledged that they suffered from some conditions after their test came out positive.

*“The signs and symptoms that are on the case-based surveillance form; most of the clients didn’t have them. Let’s say I come with a cough and then there is a client who has; a fever, and general body pains but no cough. However, upon testing these two clients; the one with a cough turns out negative for COVID-19 and the one without a cough turns out positive for COVID-19. So it was difficult for this client to understand.” (Clinician)*

The inclusion of "Other" for conditions or symptoms confused people because case definitions were not readily available, so health professionals did not know what to record.

## Process challenges (30%)

The CBSR was completed by several cadres and required good coordination to exchange information through the form. The form traveled to the laboratory with the sample for a test result. However, patients needed to be attended to by clinicians while tests were still in progress, and this was difficult to do if clinicians did not have access to information from the CBSR, e.g. underlying conditions. Here is a quote that explains such situations.

*Just to add; when we filled this form, it was going to the laboratory. It was the environmental personnel that had it. The clinician who is treating the client didn’t have it. So if the client has come, explaining that he or she is diabetic and also gets hypertension drugs; even though it was indicated on the form but maybe the client didn’t come with his or her health passport or it’s blank; we couldn’t tell that the client has other conditions. When treating this client; you were treating him or her while you were blank. Not knowing his or her other underlying conditions. That is if when the client has shown up not talking. So it was up to you to start checking everything. Which was time-consuming. Unlike if we had this form at hand. We could have known that the client had these conditions and just acted on them. Other conditions, such as diabetes; the sugar levels were going up because of maybe the drugs; the dexamethasone we were giving them. Other clients were already diabetic but didn’t know. Further, other clients knew that they were diabetic, but we didn’t have the information. That is because at first, the client was coming without a guardian then it later happened that the guardians would come along. So this was also another big challenge for us clinicians. (Clinician)*

As a note of explanation, during COVID-19 guardians were not allowed to accompany patients inside the hospitals or treatment areas. Previous studies have highlighted the essential role that guardians have in hospitals in Malawi ^26^.

Those responsible for filling in certain sections sometimes left empty essential fields, causing issues for others who were responsible for continuing to fill in other sections of the form. Some would incorrectly fill in sections designated for other cadres, e.g., environmental officers filling in sections meant for laboratory technicians, such as indicating the type of test to be conducted. The respondents were not sure of the reasons behind this behavior, whether it was due to a lack of proper training, forgetfulness, or simply overlooking the designated responsibilities.

*“I remember I mentioned something about, “Who was meant to fill the form?” On that issue, we approached our seniors in the Environmental Health Office, who clarified it to us. Remember I mentioned that we were just tossing the form to each other. We could divide the work by saying, “I will just be attending to the clients and the other personnel should be handling the case-based surveillance form.” Then later we were told, “No, there is this section that you Clinicians are supposed to fill.” However, the challenges were still there in terms of the shortage of staff. Sometimes you were alone and you could in turn fill out the whole form by yourself. Other Environmental Health personnel were not familiar with the form. So you were still filling the whole form by yourself. However, we reached out to those in authority to enlighten us.” (Clinician)*

Sometimes, important information was omitted. For example, the fields for "Date specimen collected” and “Date specimen sent to the laboratory" were often left blank by the environmental health officers who assumed laboratory personnel would fill it. This resulted in difficulties in determining the actual collection and arrival dates of test samples.

*“Apart from what my colleagues have said, the challenge I encountered had to do with the workload. It could happen that the field that says, “Date specimen collected, and date specimen sent to the laboratory” was left blank because our colleagues from the environmental(health) thought that the laboratory personnel was supposed to fill it. You would find that part has not been filled but the sample came maybe on Friday night to be processed on Monday. So it was difficult for you to know when the sample was collected and when it got to the laboratory.” (Laboratory Technician)*

To address these challenges, participants expressed the need for guidelines and clarification on the roles and responsibilities of each department, particularly in the context of data reporting and the overall management of case-based surveillance forms.

Conflicts also arose between data captured via the CBSR and via other registries, such as laboratory forms. The transfer of client details between forms necessitated understanding different formats, deciphering the handwriting of others, and resolving conflicts caused by the use of unwritten conventions, e.g., in the order of first names and surnames.

Missing data was also caused by practical aspects such as different priorities. To quickly obtain test results, laboratory technicians placed more importance on capturing essential demographic data, such as the individual's name, date of birth, and sex. As a result, forms filled out by laboratory personnel frequently had gaps or omissions of other essential information such as symptoms or underlying conditions.

High workloads in busy health facilities also contributed to difficulties in accurately completing the form, personnel noted the impact of small fields, especially in hectic environments.

*“The other issue is what has already been said. That, it was too involving. For instance, there was a scenario when COVID-19 had reached its peak; you could screen hundreds to two hundred clients alone. So you were supposed to fill in the form for every client and at the same time there were also other forms you were supposed to be filling in to clear the clients and whatnot. Thus, it was too involving. (Clinician)”*

Laboratory technicians faced challenges due to limited access to data and internet connectivity. As such, submitting test data on weekends, particularly on Saturdays and Sundays, proved to be difficult due to additional work responsibilities and time constraints. This often resulted in missed submission deadlines, and to the buildup of large backlogs of cases. This meant weekly reported numbers became inaccurate over time. In the absence of adequate resources, personal phones were used for sending test results to patients or sending numbers to district focal persons, for contact tracing and following up, and for collecting information about case outcomes.

Additional complications resulted from the use of three copies of the CBSR: the original, and two copies, one yellow and one blue. The original was sent to the laboratory and was meant to be a complete record for a case. However, the copy kept at the facility was almost always incomplete, for example, it rarely recorded test results or case outcomes. This confusion in record-keeping led to a strenuous effort to reconcile information between different offices. Later on, a decision was made to appoint only one custodian for the original copy, e.g. in Lilongwe, that being the IDSR office. This led to some improvements in the recorded data but added extra activities to ensure the flow of data.

*“Another big challenge that I experienced using the case-based surveillance form, had to do with the system of inputting the data. The form has duplicate copies. So, as I said, we divided the work. Different cadres were handling the form. For instance, at isolation camps, the “Environment Health” personnel were just filling their section and then leaving out the Laboratory Technicians section. They take out the first copy and then give it to the client. Therefore, the remaining two copies of the form did not capture the results from the Laboratory. The first copy was the only form capturing the results. Of which, it was easy for it to go missing. If that happens, it means that the two remaining copies would not have the results indicated.” (Laboratory Technician)*

Calculating accurate aggregate test results nationally were also impeded by the coordination and linkages of data between multiple stakeholders who operated independently or privately and did not always share data.

## Training issues (19%)

Training on form completion took place on the job, and this was considered a major cause of data discrepancies, as were other factors regarding data management processes.

*“At first it was kind of challenging since we didn’t undergo any training on how to fill in the forms. It was more like on-the-job training. As we were working; we were also getting acquainted with the case-based surveillance forms. It was kind of tough at first because the information seemed to be a lot. So, you were considering the client and the condition he or she is in. If you asked them the questions, you would see that some of the clients are failing to explain what the problem is; or maybe just asking their birth year; some didn’t know their birth year, and where they’re coming from.” (Environmental Health Officer)*

Some formal training was provided in the early stages of the COVID-19 pandemic but was limited to a select number of cadres from some departments and did not cover the content and guidelines for completing CBSRs and line-lists. The nature of COVID-19 meant that many more people were later involved in the data collection including untrained interns. As the form encompassed several different diseases, there was a need for training and orientation for all staff members on data capture, reporting, data management, and the meaning of fields on the form.

*“My perception about this case-based surveillance form is that it seems it had all the parameters which were supposed to be captured. However, it had some other parameters which were supposed to be explained very well. So that the one who is filling it should understand the meaning of these other words that are on the case-based surveillance.” (EHO)*
*I think the type of training needed was; for instance, the personnel from “PHIM” to propose a place, so that people could gather there. Then thoroughly explain to them how to use the case-based surveillance form. That is because I can come and explain something different about the form. I will tell you that, this is the way we fill out the form, since it just on-the-job training. That’s how we were doing it, right? To say, “How do we do this?” Then someone would explain, “This is how we fill the form. If you get to this part, send the form to the Laboratory and so forth.” So we are different people. I can tell someone the way I have understood how we should handle the form. Some other person will also go and tell another person the way he or she has understood how to use the form. As it goes, the way you’re filling the form would change, (Laboratory Technician)*
*“I think the training is needed. For instance, we attended a certain training, and the persons there were debating that, “The case-based surveillance form is supposed to be at the Laboratory. Others said that the form is supposed to be at the “OPD” and other people said that the form is supposed to be with the data personnel.” So because there were some errors, people were contradicting each other. On the other hand, if we had an orientation; to maybe let the health workers know that, anyone can fill the case-based surveillance form or that this section was supposed to be filled by the Laboratory Technicians and whatnot. Then that confusion could have been cleared from the very beginning. Furthermore, some other personnel didn’t know what some of the abbreviations on the form meant. That what is Participant Three was saying; that we were just enquiring from each other. So if the person responding tells you the wrong things. That meant the whole form, will be filled the wrong way. However, at the training, you emphasize the importance of filling out the form. With this form, that emphasis was not there. The form was regarded as any other form that we usually fill. Thus, even leaving the form blank was not really a problem because we didn’t even know how the forms were supposed to be filled. Further, to say; was it important to fill the whole form or not? So, there is really a need to sit down and have the training.” (Clinician)*

The training must also be multidisciplinary so that the processes are understood by all cadres. Clarity on responsibilities, workload, and remuneration was also mentioned by many of the respondents.

## Language issues (2%)

Language issues were considered important. While 2% is the proportion in terms of the number of mentions the issue received, the issue is of notable importance. The form was in English. Asking about underlying conditions or diseases in English posed comprehension issues for some clients.

*“Now even the ones who were handling this form also had problems because they couldn’t interpret some of these symptoms in Chichewa. So on its own that was also challenge due to language. That is because those people were not medical personnel. They couldn’t interpret for instance, fatigue, shortness of breath, skin rash, chronic diseases. These things were difficult for them to interpret in Chichewa.” (Clinician)*

Well-thought-out translations are essential. Direct translations from English may introduce additional misconceptions, e.g., “underlying conditions” are often translated as “matenda amgonagona”, which is usually interpreted as sexually transmitted diseases. This could have been the reason why clients did not see the relevance to COVID-19 of being asked about underlying conditions when this was deemed to refer to something that they found hard or embarrassing to disclose. Such language misunderstanding could have caused some of the instances in which underlying conditions field remained empty on many forms. There was a need for interpreting medical terms in Chichewa, and this proved difficult to do on the spot: *“Our clients had difficulties differentiating chest pains and shortness of breath. You could ask the client; are you feeling fatigued? The client responded “Yes.” Are you having chest pains? The client would also respond, “Yes.” Are you having shortness of breath, the client would again respond, “Yes.”*

## Privacy, ethical, and cultural issues (4%)

The clients who received COVID-19 tests were not given the same privacy as other clients, e.g. those who receive human immunodeficiency virus (HIV) testing. Consultations took place in a tent or a room with many people waiting in proximity: *“On the underlying conditions field, for instance, when you’re entering a consultation room and let’s say; two or three clients enter the room at once, that means a client cannot open up about his or her HIV status”.* Privacy concerns and societal stigma around questions related to pregnancy, HIV status, and mental illness lead to information gaps as clients withheld sensitive details.

*“On line listing, especially on the “Health conditions;” some health conditions are very sensitive. For example, I wrote a lot of line lists, but I never heard a client say, “I’m HIV positive or I have Tuberculosis.” Some questions are very sensitive and the clients were hiding information. Further, for example, mental illness, can a person say that I have a mental illness problem.”* The “trimester” field surprisingly caused some challenges and highlighted the importance of being aware of social norms and context: *“Sometimes the client was shy to mention how many months her pregnancy is for. Therefore, we were just leaving it blank”.* When surveillance took place in schools, asking this question also caused problems. This observation serves as a compelling example of the intricacies involved in data collection and the significance of considering contextual factors. The stigma associated with COVID-19 made it difficult for health workers to get close to clients and obtain information. Some health workers were initially afraid of contracting COVID-19, so they sometimes filled out the forms quickly. The presence of fields requiring reporting personnel to add their name and phone numbers created uncomfortable situations when customers who were given a copy of the form could later call privately to inquire about their tests or other issues. Ethical issues and data reliability were caused by situations in which the respondents were minors (e.g., testing in schools, information about medical history was known only to parents/guardians) or were situated in refugee camps or in prisons. In some of these instances, interpreters had to be used; thus, the respondents may have had reasons to withhold information.

## Results from interviews

The scope and purpose of electronic systems used in Malawi varied, which meant that their utilization for national COVID-19 surveillance was poor; instead, paper-based processes were preferred. This contributed to the overall disparity in data collection efforts, leading to challenges in the reconciliation and integration of various data sets collected in public and private facilities. Informants revealed that some notable changes in processes and use of the form occurred due to the overwhelming nature of COVID-19 surveillance, which took place on a large national scale.

The first type of change was the use of a single form, the CBSR, considered to be logistically easier to use on the field and less expensive to produce than using multiple forms. The CBSR was initially used for cholera surveillance and became bulkier with the addition of other COVID-19-specific fields. Problems with the form design and its use had the potential to become more pronounced during COVID-19 because of the large number of cases. Data quality issues were also harder to fix given the increase in the amount of data collected via the form.

To address the issue of data loss due to the use of three copies of the CBSR form, certain steps were taken, such as avoiding the movement of the original CBSR between the IDSR office and the laboratory. However, this led to other types of problems. As the volume of tests increased, the data entered in the Laboratory Management Information Systems only captured essential data points related to a test and did not include most of the fields present on the CBSR forms such as underlying conditions or symptoms. Some of this data was never recovered, and many of the logbooks stored were lost due to damage by termites.

Other changes were made in terms of coordination among cadres. The form included clinical examination, laboratory information, and case outcome information. These sections were intended to be filled by three different types of cadres: environmental or health surveillance assistants, clinicians, and lab officers. However, this approach was only adopted in practice during the first wave. Later, it became impractical due to staff shortages and an increase in workload. As a result, numerous data fields were left unfilled, as coordination among cadres was tricky. Subsequently, the responsibility for data collection shifted entirely to environmental health officers, who completed all sections of the form.

Those interviewed raised the need for comprehensive training covering the entire form for all cadres. Training in data recording became even more important due to reliance on HSA interns for surveillance and mass testing. The use of interns became necessary for COVID-19 due to the large volume of testing needed to be carried out. Interns received summary instructions on what to do and how to fill out forms. They lacked formal training, and it could be argued that this impaired the effective management of COVID-19. The novelty of the disease further complicated the situation. According to District Environmental Health Office Case Management Lead, a limited number of individuals, such as only 16 out of 500 in Lilongwe, received formal training on COVID-19 management.

It was recognized at the national level that there was a need to address data challenges from the ground up and to understand structural issues before moving into complex tasks such as running predictive analysis *“because the data flow was not much guided (...) since we could not have information about disparities in those forms…we have been managing without analysis.”*

## Document analysis

From interviews and document analysis, we derived roles and responsibilities for key cadres involved in COVID-19 surveillance ( Table 6). We noted their involvement in completing CBSR forms and other COVID-19 data collection tools and what was the required coordination among cadres.

## Form audit

To obtain a measure of data completeness, we randomly audited 47 completed case-based surveillance books from various locations in Lilongwe, such as district hospitals, schools, and prisons. Books contained about 100 CBSR copies. We found that most of the forms were filled in by environmental health officers (96%), and a few by lab officers (4%). In all audited books, the case outcome fields were blank, and 96% had the test outcome blank.

To further understand the problems with the empty fields, we conducted a separate audit of 122 originals completed in 2022. We examined the laboratory and case outcome/classification sections. All the audited originals were found to have blank case outcomes and final classification. There was an improvement in the recording of test results, 74% of the original forms recorded test results. This happened when tests were based on rapid antigen kits that took about 15 min to arrive at a result and could be done on site. The forms recorded less than 40% of the required data. The audit indicated that while the form was comprehensive in terms of the fields required by authorities to support informed COVID-19 public health decisions, the disconnect between the data needs and the processes available to support its collection acted as a barrier to the effective utilization of the forms.

## Conclusions and recommendations

In this study, we report on the utilization of CBSRs and line lists for compiling case-based COVID-19 national data in Malawi. The material we collected through FGDs, interviews, and form audits was rich, and we extracted themes that were relevant for enhancing the design of the data collection tools and processes. We can conclude that optimal utilization of surveillance forms required further training, language considerations, and improvements in design. This becomes even more pressing when data collection is intensive, and the use of data capture tools relies on interdepartmental coordination. The high workload during the COVID-19 pandemic, lack of suitable training, and unclear processes contributed to difficulties in accurately capturing data on forms and line lists causing data inconsistencies and gaps. Participants proposed some solutions such as the use of separate forms for different diseases, the introduction of specialized data handling personnel, and better access to guidelines on roles and processes. Staff dedicated to data tasks, akin to medical scribes ^27^, could be the missing link in ensuring data alignment between facilities and districts, better data entry and utilization. There is also a need for establishing and maintaining a library of standard templates and completion manuals ^28^. The availability of forms and guidelines in local languages could help respondents communicate better and more accurately their conditions and symptoms.

This study involved health surveillance professionals in two cities of Malawi: Lilongwe and Blantyre. The participants represented an important sample because most COVID-19 cases were recorded in these two cities and staff at these locations have better access to formal training than colleagues in other parts of the country. Our study emphasized the need to revisit both processes and form design and to rethink the type of training that is available to health professionals at the point of care. The need for better healthcare training in Malawi, especially for community health workers, has been investigated by other authors ^25, 29– 31^. Our study is unique because we looked at concrete evidence of how, despite significant efforts being made, inadequate training and poor access to knowledge resulted in the inability of healthcare professionals to generate quality data needed for decision-making. Our study invited participants to reflect on how the processes they followed and the forms they filled in were linked to data quality issues and decision-making. We hope that this study brings fresh evidence to the current situation and helps formulate solutions to improve the quality of the data collection tools and processes for disease surveillance and other emerging/re-emerging diseases in the country.

## Abbreviations

COVID-19     Coronavirus Disease 2019 caused by SARS-CoV-2

IDSR              Integrated Disease Surveillance and Reporting

HSA               Health Surveillance Assistants

EHO               Environmental Health Officers

CBSR             Case-Based Surveillance Reporting

PHIM             Public Health Institute of Malawi

FGD               Focus Group Discussions

HIV                Human Immunodeficiency Virus

## Ethics and consent

The project was sponsored and approved by the Malawi National Health Sciences Research Committee protocol #21/03/2669 issued on 11 ^th^ March 2021 and renewed on 4 ^th^ March 2022 and by local research ethics of the Lilongwe District Health on 2 ^nd^ May 2023 and the Blantyre District Health Research Committee on 24 ^th^ July 2023 permitted the team to conduct the study at the research sites in Lilongwe and Blantyre. Participants were required to provide written informed consent, which was collected by trained facilitators, and all data and documents were kept under the custody of the principal investigator in a locked cabinet, located in a restricted-access area.

## Data Availability

Open Science Framework: Insights into COVID-19 Data Collection and Management in Malawi: Exploring Processes, Perceptions, and Data Discrepancies. https://doi.org/10.17605/OSF.IO/C5SFE ^6^. The dataset contains focus group discussion guides, interview guides, consent forms, supplementary materials data collection tools, a copy of the Malawi Case Based Surveillance Form, codes used in thematic analysis, and results summaries. Data are available under the terms of the Creative Commons Attribution 4.0 International license (CC-BY 4.0). The audio and raw data of the FGD and interviews cannot be shared due to security and privacy concerns. Audio recordings contain various identifiable information that may be used to identify a participant. In addition to the words that a person expresses vocally, vocal characteristics they provide - their speech pattern, accent, vocabulary – may be sufficient to determine their identity. Unidentified and anonymized text transcripts are available to bona fide researchers and students at universities and research institutes. Interested parties should contact the first author Amelia Taylor through ataylor@mubas.ac.mw in the first instance, quoting the paper title. After a discussion of the data needed, a signed data transfer agreement would be required.
