# Supplementary material for: Insights into COVID-19 data collection and management in Malawi: exploring processes, perceptions, and data discrepancies
Source: Wellcome Open Res. 2024 Apr 24;9:217. [Version 1] doi: 10.12688/wellcomeopenres.21131.1 (PMC11303952; doi:10.12688/wellcomeopenres.21131.1)
Supplement: Peer review history

# Reviewer report: Reviewer response for version 1

**DOI:** 10.21956/wellcomeopenres.23375.r87283

**Published:** 2024-08-06

**Authors:** Patrick Mapulanga

**Affiliations:** 1 Kamuzu University of Health Sciences, Lilongwe, Malawi

The abstract presents a well-structured and relevant study with clear objectives and a robust methodological approach. It highlights significant findings related to data quality issues in COVID-19 surveillance in Malawi. However, it could benefit from a more balanced inclusion of quantitative data, broader participant selection, and more detailed exploration of less frequently mentioned issues. The conclusions could also be enhanced with specific recommendations to provide clearer guidance for future improvements.

 The introduction provides a thorough and well-supported background on the importance of surveillance systems for COVID-19 in Malawi, detailing the challenges and adaptations made. It identifies data quality issues as a significant problem, justifying the need for the study. However, it could be improved by being more concise, explicitly stating the study’s objectives, and making the text more accessible to readers with varying levels of prior knowledge. Balancing the level of detail and emphasizing the broader implications of the study would also enhance its effectiveness.

 The methods section outlines a comprehensive and well-structured approach to capturing the experiences of individuals involved in COVID-19 data collection in Malawi. It strengths include detailed participant selection, the use of multiple data collection methods, and adherence to ethical practices. However, it could be improved by addressing potential biases, expanding the sample size and geographic range, providing more detailed information on data analysis techniques, and better integrating quantitative data. Enhancing these areas would increase the overall robustness and credibility of the study.

 The results section could benefit from a more balanced inclusion of quantitative data, broader participant selection, and more detailed exploration of less frequently mentioned issues.

 While the form is comprehensive and familiar to health professionals, its length, complexity, and lack of clear guidance result in significant challenges. These issues lead to incomplete and inaccurate data, which undermines the form's effectiveness for surveillance and contact tracing. Addressing these weaknesses through better design, training, and client engagement would improve data quality and usability.

 The analysis provides valuable insights into the challenges faced by participants in filling out the CBSR, highlighting critical areas for improvement in form design, process coordination, and training. However, it would benefit from more quantitative data, specific recommendations, and a more focused approach to addressing the identified issues. By addressing these weaknesses, the analysis could offer a more comprehensive and actionable set of findings for improving the CBSR process.

 Overall, the study presents a thorough analysis of the challenges faced in COVID-19 data collection in Malawi and provides practical recommendations for improvement. However, it could benefit from a broader geographic scope, more quantitative data, and a deeper exploration of electronic systems and alternative solutions. The findings are insightful and relevant, offering a valuable contribution to the understanding of data collection challenges and potential solutions in the context of a public health crisis.

Is the work clearly and accurately presented and does it cite the current literature?

Yes

If applicable, is the statistical analysis and its interpretation appropriate?

Partly

Are all the source data underlying the results available to ensure full reproducibility?

Yes

Is the study design appropriate and is the work technically sound?

Yes

Are the conclusions drawn adequately supported by the results?

Yes

Are sufficient details of methods and analysis provided to allow replication by others?

Yes

Reviewer Expertise:

implementation science

I confirm that I have read this submission and believe that I have an appropriate level of expertise to confirm that it is of an acceptable scientific standard, however I have significant reservations, as outlined above.

# Reviewer report: Reviewer response for version 1

**DOI:** 10.21956/wellcomeopenres.23375.r82984

**Published:** 2024-07-31

**Authors:** Francis Levira

**Affiliations:** 1 Ifakara Health Institute, Ifakara, Morogoro Region, Tanzania

The manuscript focus case-base surveillance, an important and underdeveloped area in most developing countries. The findings well contribute in further development of surveillance system in developing countries in the context of weak electronic data collection systems, unplanned neighbourhoods, and limited health care workforce. The article need some major revisions in terms of focus (less is always more) and logical flow of thoughts.

 Your objectives are not clear organised in the manuscript. It is important to get everything aligned.

**Abstract:**

 “We collected participants’ views on the effectiveness of surveillance forms in collecting the intended data, as well as on the data collection processes and training needs”

**Main paper:**

 This study aimed to gain insights into the operational aspects of COVID-19 data management, understand health worker perspectives on surveillance data, and identify factors influencing data accuracy and consistency within the district’s reporting system.

**Conclusions and recommendations:**

 “In this study, we report on the utilization of CBSRs and line lists for compiling case-based COVID-19 national data in Malawi”

- I am not seeing your objectives being clearly answered in your conclusion. Your conclusion and recommendations should be revised and reflect key issues to be addressed.
- You may also need to do a small comparative narrative of other case-base surveillance tools and data flow used in other countries (WHO standard?) to put your findings in perspective.
- In general your study is spread over so many aspects of data and it would be very important probably to well structure your study as mixed method or keep it qualitative or quantitative. If need be, you may develop another paper on data quality. No statistical analysis has been conducted to answer the part “and identify factors influencing data accuracy and consistency”. Document analysis and form audit part are not well connected or articulated to the paper.

**Other issues:**

1. Use diagram to describe data collection process and flow. It will make the paper and method clearer because data is collected from multiple tools (CBSR and line lists-hospital notes?), format (hard copies and digital), and locations. See this reference ( https://www.ncbi.nlm.nih.gov/pmc/articles/PMC9009682/): Umeozuru CM *et al.* (2022 ^1^)
2. The introduction section is long and information that best fit the method section. Revise this part. Focus on COVID CBS tools applies in other context and related studies focusing on health worker perspectives in Malawi and beyond.
3. Shorten your article and reduce the burden to the reader…there are text which are not necessary and do not add value!
  This part is repealed in methods section:
  “In Malawi, case surveillance data was first collected by facilities and thereafter sent to the district levels, where IDSR focal persons, responsible for its quality, aggregated it and sent it nationally to the Ministry of Health for analysis and public dissemination. For COVID-19, cases were recorded using the Case-Based Surveillance Reporting form (CBSR) and line lists6. The CBSRs were completed in hard copies, and line lists were both digital and in hard copies.”
  “The use of MAXDQA allowed us to man- age all our resources in one place, to define and apply codes efficiently to paragraphs and sections of documents, and to obtain insights into the data based on applied codes. MAXDQA is a paid software, but several free alternatives exist, the clos- est in terms of features to MAXDQA being QualCoder24.”
4. The study was part of a project conducted in close collabora- tion with the Ministry of Health through the Public Health Institute of Malawi. The scope of the project was to under- stand the national COVID-19 data collected via IDSR surveil- lance in Malawi.
5. Shorten Table 1, remove, Age ranges, Years of Participation in COVID-19 data collection, Highest Number on average of cases participants filled each week, n
6. Remove table 2, 3, and 6 if necessary, put it in appendix
7. Results from interviews part has less (only one) quotation. You may consider adding few more.

Is the work clearly and accurately presented and does it cite the current literature?

Partly

If applicable, is the statistical analysis and its interpretation appropriate?

Partly

Are all the source data underlying the results available to ensure full reproducibility?

Yes

Is the study design appropriate and is the work technically sound?

Partly

Are the conclusions drawn adequately supported by the results?

Partly

Are sufficient details of methods and analysis provided to allow replication by others?

Yes

Reviewer Expertise:

Public health, epidemiology, data management, surveillance

I confirm that I have read this submission and believe that I have an appropriate level of expertise to confirm that it is of an acceptable scientific standard, however I have significant reservations, as outlined above.

## References

[1] : Performance of COVID-19 case-based surveillance system in FCT, Nigeria, March 2020 -January 2021. *PLoS One* .2022;17(4) : 10.1371/journal.pone.0264839 e0264839 10.1371/journal.pone.0264839 35421123 PMC9009682

# Reviewer report: Reviewer response for version 1

**DOI:** 10.21956/wellcomeopenres.23375.r82977

**Published:** 2024-05-22

**Authors:** Sarah Markham

**Affiliations:** 1 King's College London, London, England, UK

This is a fascinating study highlighting the integral connection between data quality and the design and utilization of data collection forms. It is of significant public health relevance and captures practicable learning. I think this study will be a valued addition to the research literature, in addition to informing future policy and practice in the data collection and management regarding pandemics.

Is the work clearly and accurately presented and does it cite the current literature?

Yes

If applicable, is the statistical analysis and its interpretation appropriate?

Yes

Are all the source data underlying the results available to ensure full reproducibility?

Yes

Is the study design appropriate and is the work technically sound?

Yes

Are the conclusions drawn adequately supported by the results?

Yes

Are sufficient details of methods and analysis provided to allow replication by others?

Yes

Reviewer Expertise:

Public health and epidemiology

I confirm that I have read this submission and believe that I have an appropriate level of expertise to confirm that it is of an acceptable scientific standard.
